# Supplementary material for: Multiple Components of Protein Homeostasis Pathway Can Be Targeted to Produce Drug Synergies with VCP Inhibitors in Ovarian Cancer
Source: Cancers (Basel). 2022 Jun 15;14(12):2949. doi: 10.3390/cancers14122949 (PMC9220887; doi:10.3390/cancers14122949)
Supplement: Supplementary file 1 [file cancers-14-02949-s001.zip › cancers-1737744-supplementary.pdf]

Title: Multiple components of protein homeostasis pathway can be targeted to produce drug synergies with VCP inhibitors in ovarian cancer.

Prabhakar Bastola,[1] Gary Leiserowitz,[2] and Jeremy Chien*[2,3]

[1]Department of Pharmacology, Toxicology & Therapeutics, University of Kansas Medical Center, Kansas City, KS 66160

[2]Department of Obstetrics and Gynecology, University of California, Davis, CA 95817

[3]Department of Biochemistry and Molecular Medicine, University of California, Davis, CA 95817.

Figure S1

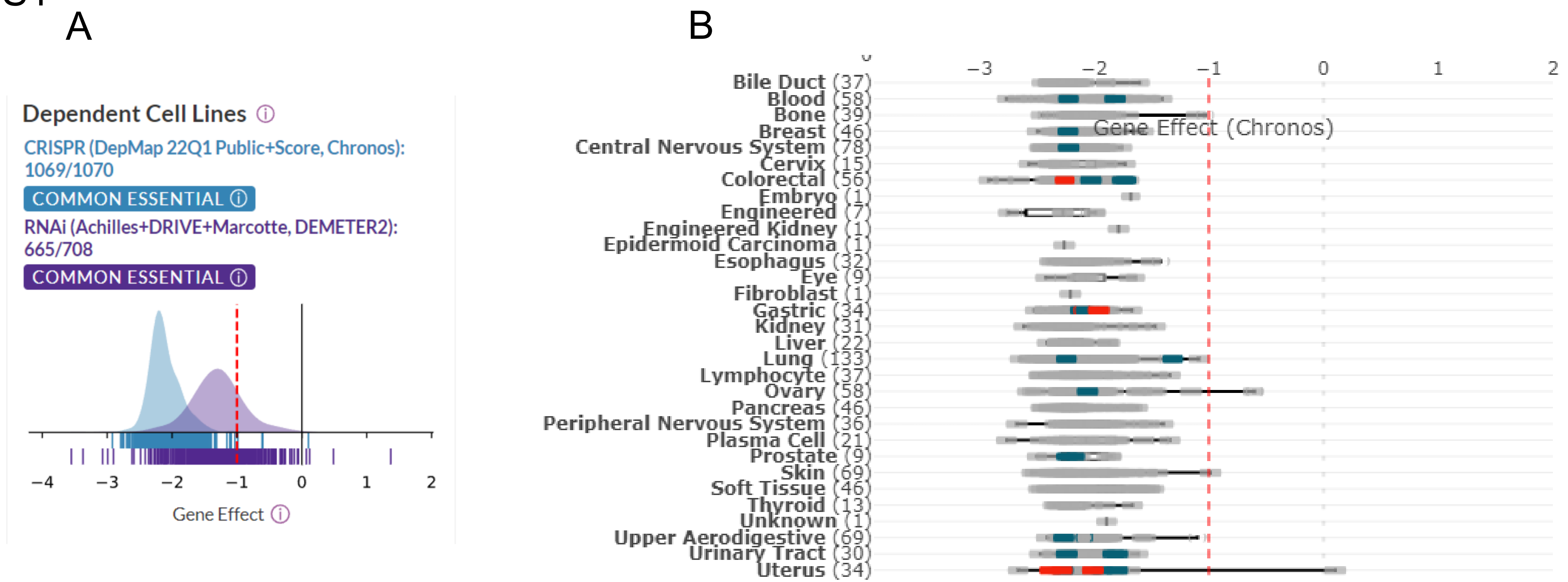

A:Gene effect depicted from CRISPR and RNAi screens . A lower gene effect score suggest that the gene is more likely to be essential in the tested cell line. Score of 0 represents non-essential, while score of -1 represents a median for all common essential genes. B: Gene effect score depicted from CRISPR screens in different cancer cell lines, including ovarian cancer cells (ovary). Both data sets was obtained from the DepMap Portal on April 02, 2022.

Figure S2

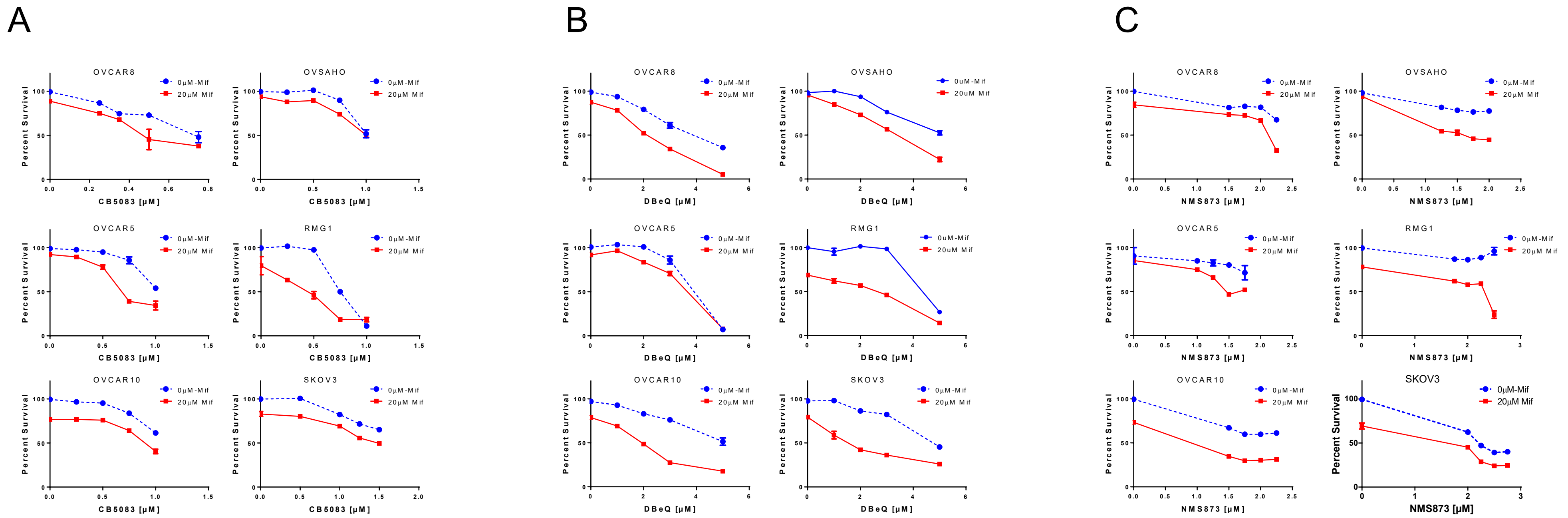

A: Dose response following treatment with vehicle, indicated concentration of CB-5083 with or without 20 μM mifepristone in indicated ovarian cancer cells. B: Same as A, but cells were incubated with indicated concentrations of DBeQ instead of CB-5083. C: Same as A, but cells were incubated with indicated concentrations of NMS-873. Dose-response curves were generated using GraphPad Prism. Every point in the dose-response curve represents Mean ± SEM taken from three technical replicates for all cell lines.

Figure S3

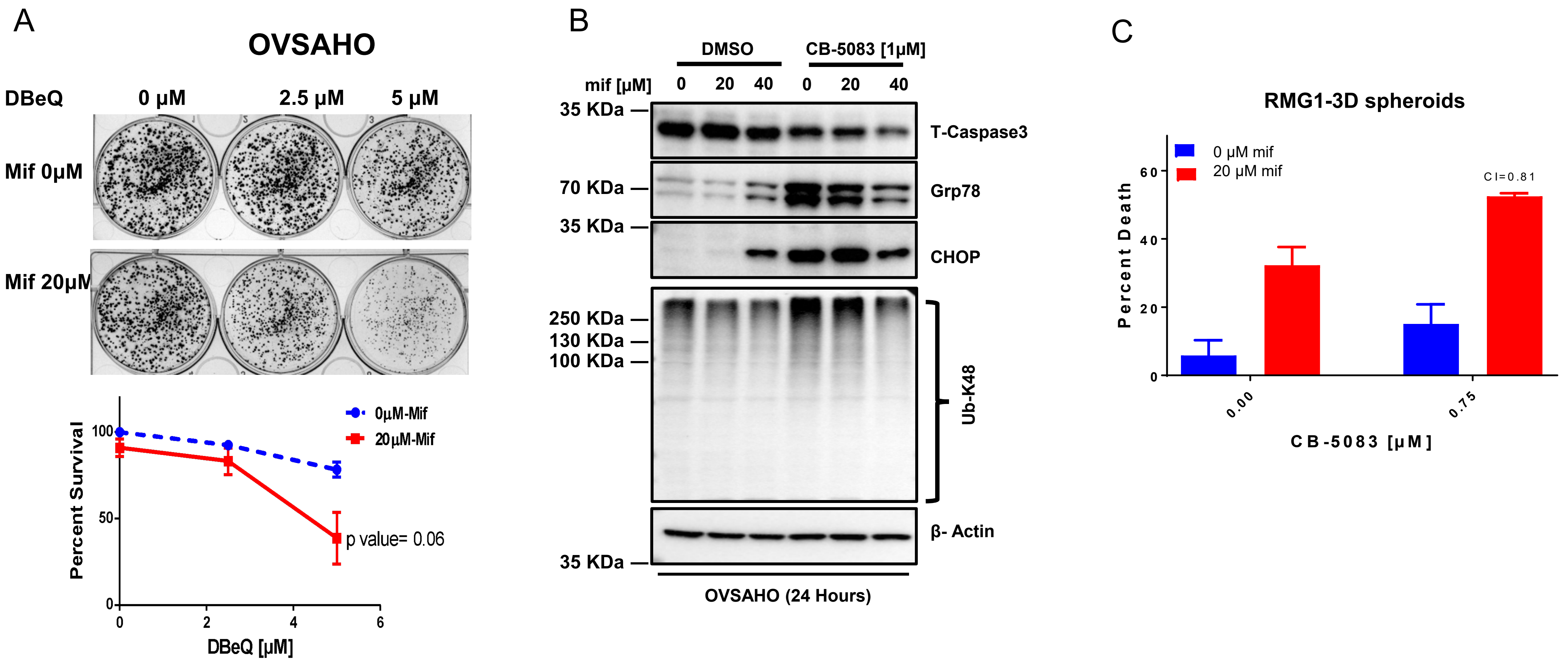

A: OVSAHO cells treated with indicated concentrations of DBeQ and mifepristone for 48 hours followed by 6-8 days of recovery in regular media. Percent survival was calculated based on the number of colonies from three biological replicates. P values were calculated using the two-tailed student's t-test. B: Western blot analysis of total (T) and cleaved (Cl) caspase 3, full length (FL) and cleaved PARP1, and ER stress-related proteins at 12 hours and 24 hours after CB-5083 and/or mifepristone (mif). B. Immunoblot (IB) analysis of ubiquitinylated (K48-linked) proteins following CB-5083 or mifepristone treatment in OVSAHO cells. C: RMG1 spheroids were treated with indicated concentrations of vehicle (DMSO), CB-5083 and mifepristone for 72 hours. Percent cell death was analyzed using 3D CellTiter-Glo Assay from three biological replicates.

Figure S4

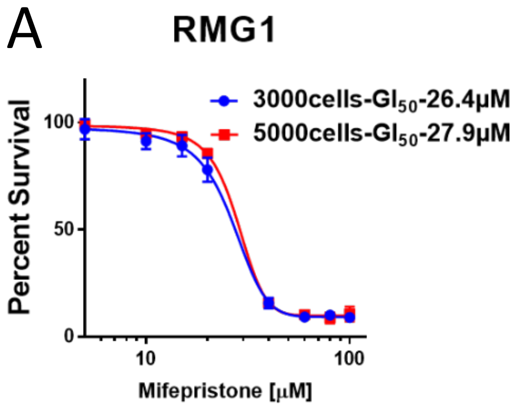

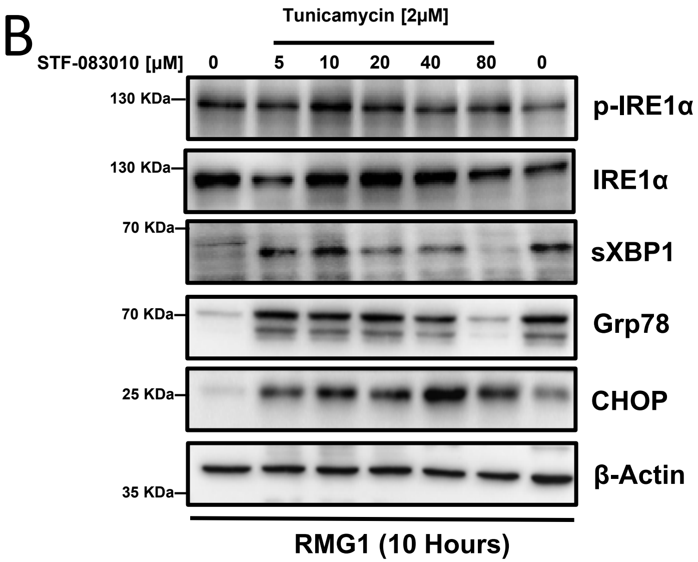

A: RMG1 were treated with increasing doses of mifepristone ranging from 10 μM to 100 μM for 72 hours. Dose-response curves were generated using GraphPad Prism based on the four parameters nonlinear regression. The curves were constrained at the top (100%) and the bottom (>0%). Every point in the dose-response curve represents Mean ± SEM taken from three technical replicates for all cell lines. B: RMG1 cells were incubated with increasing doses of IRE1α inhibitor- STF-083010 (5 μM – 80 μM) with 2 μM of tunicamycin for 10 hours.

Figure S5

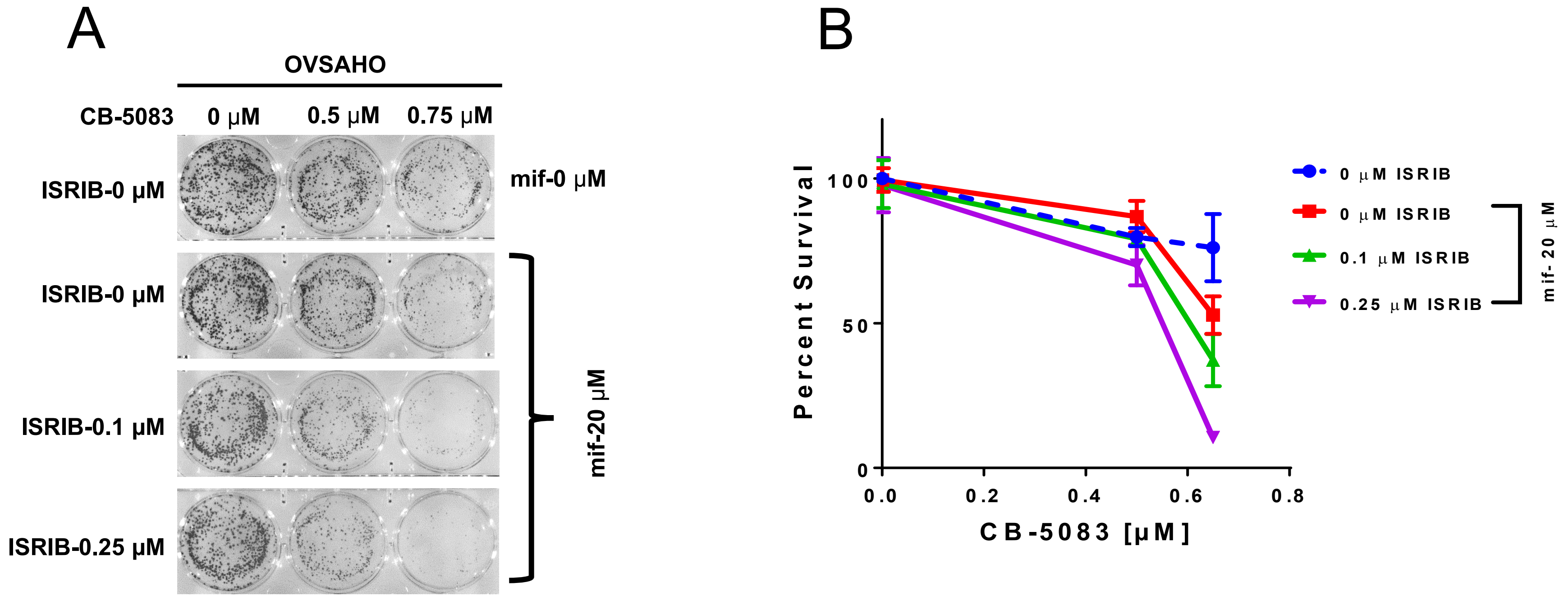

A: Clonogenic assay to assess the triple combination of CB-5083, ISRIB, and mifepristone in OVSAHO cells. B) Mean percent survival ± SEM was calculated based on number of colonies from three biological replicates.

Figure S6 – Original western blot from Figure 3

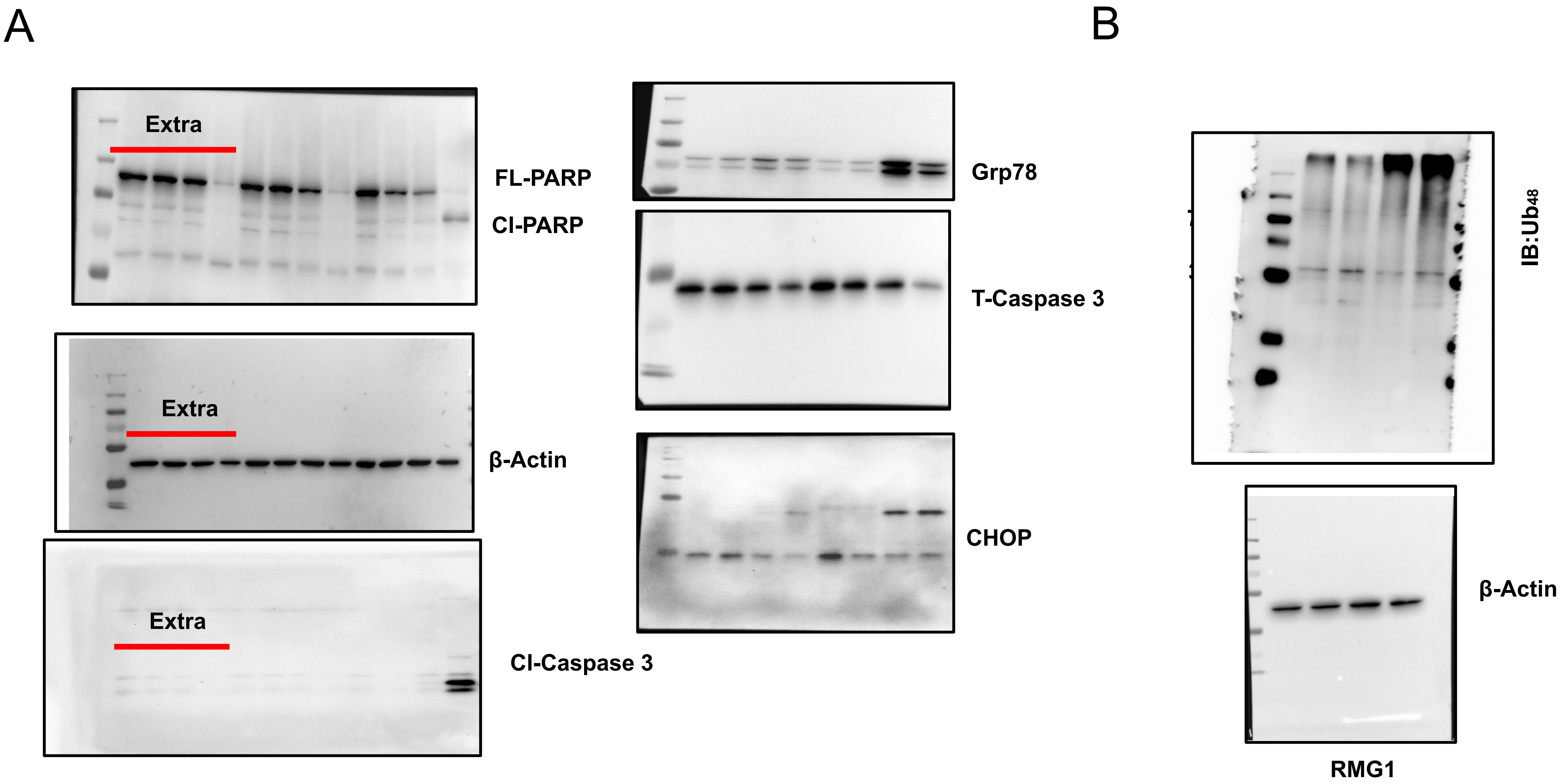

Figure S7 – Original western blot from Figure 4

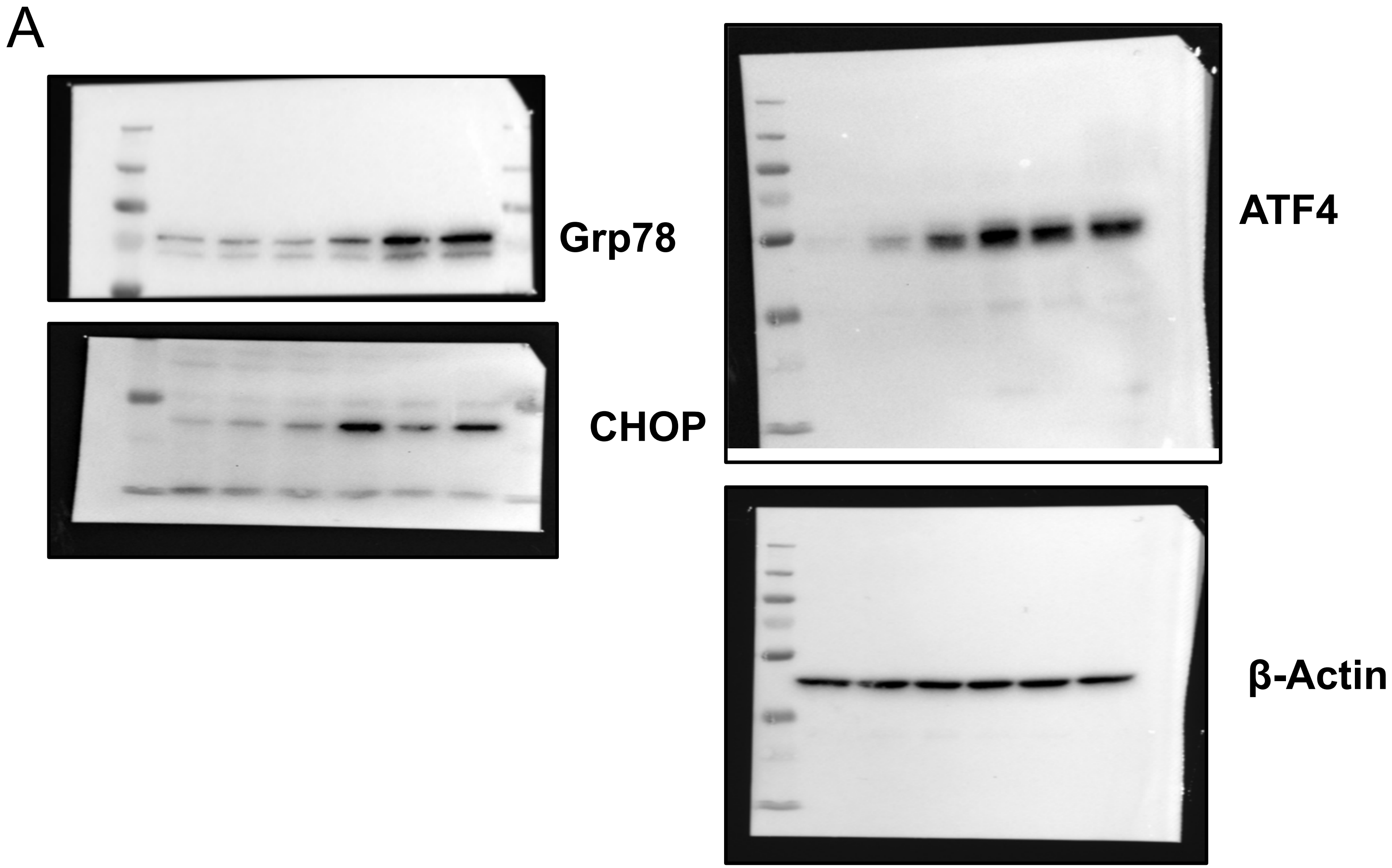

Figure S8 – Original western blot from Figure 5A

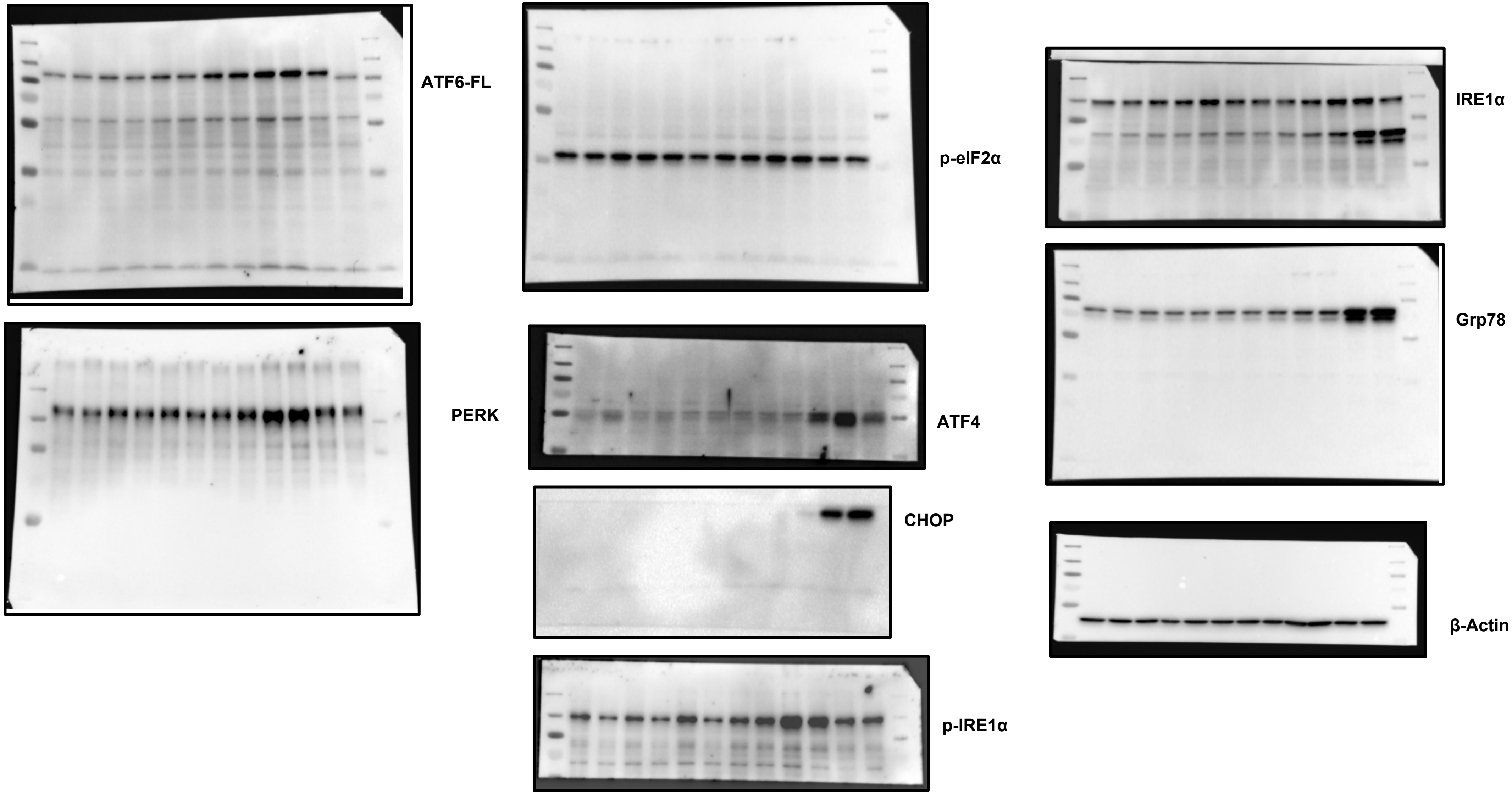

Figure S9 – Original western blot from Figure 5B

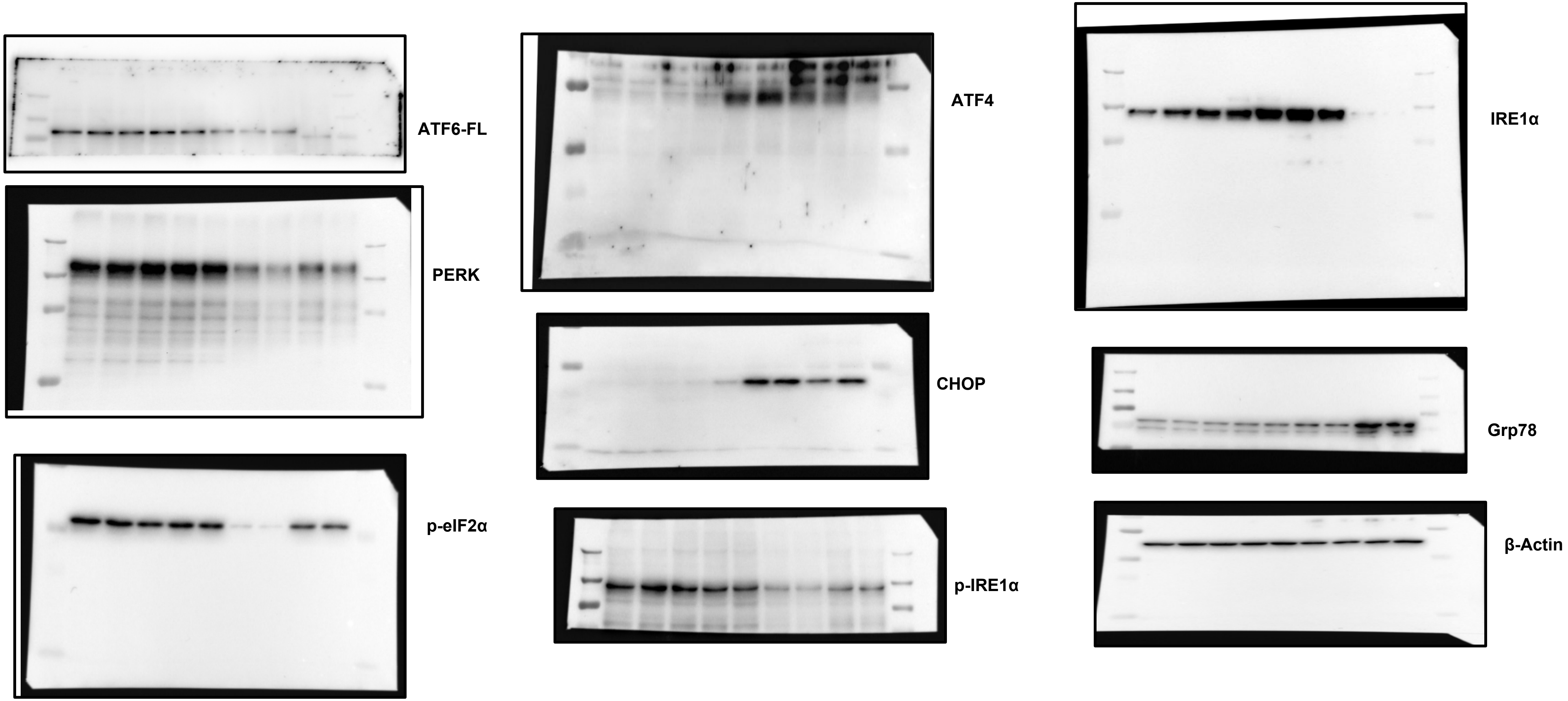

Figure S10 – Original western blot from Figure 5C

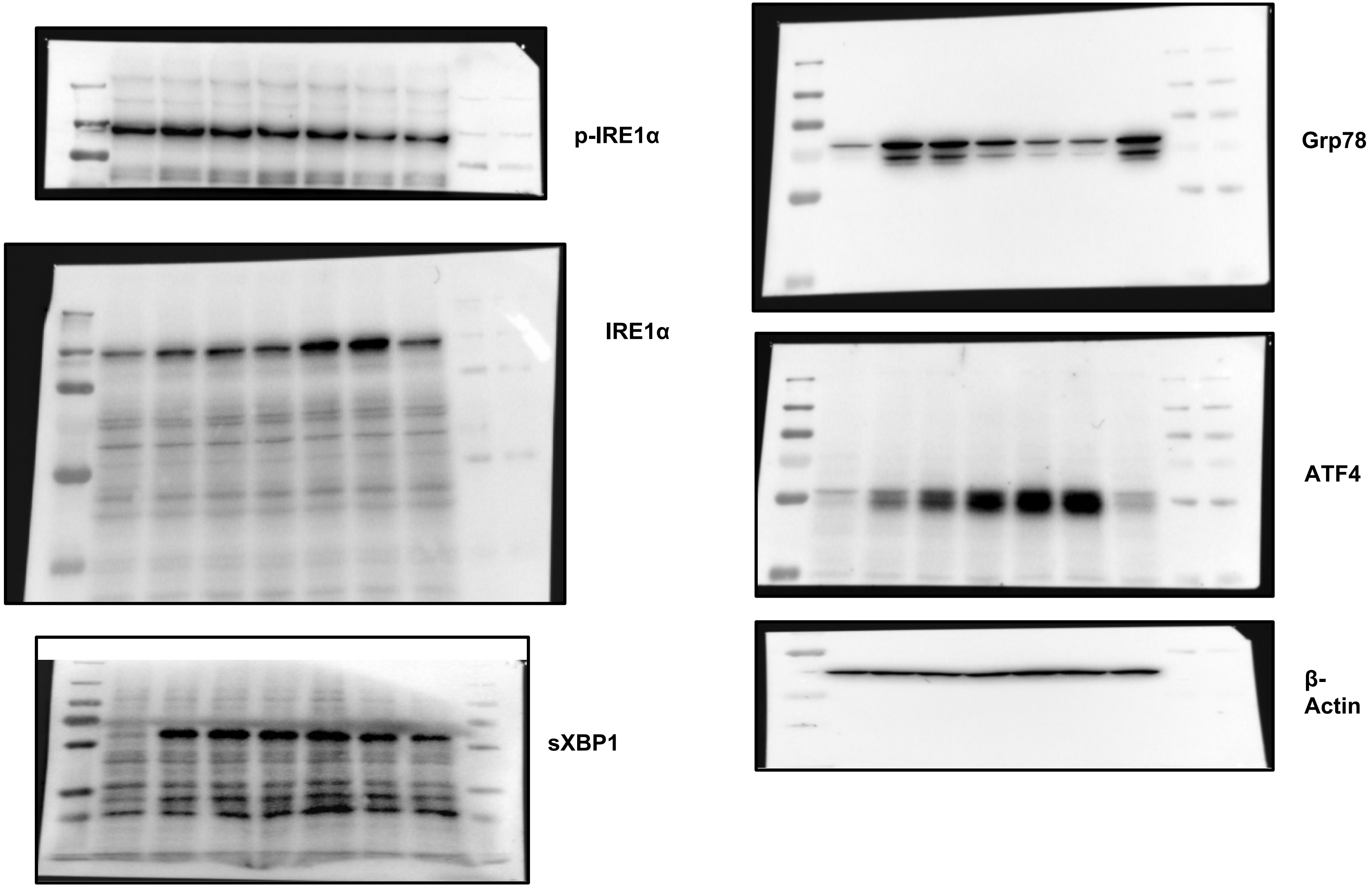

Figure S11 – Original western blot from Figure 5D

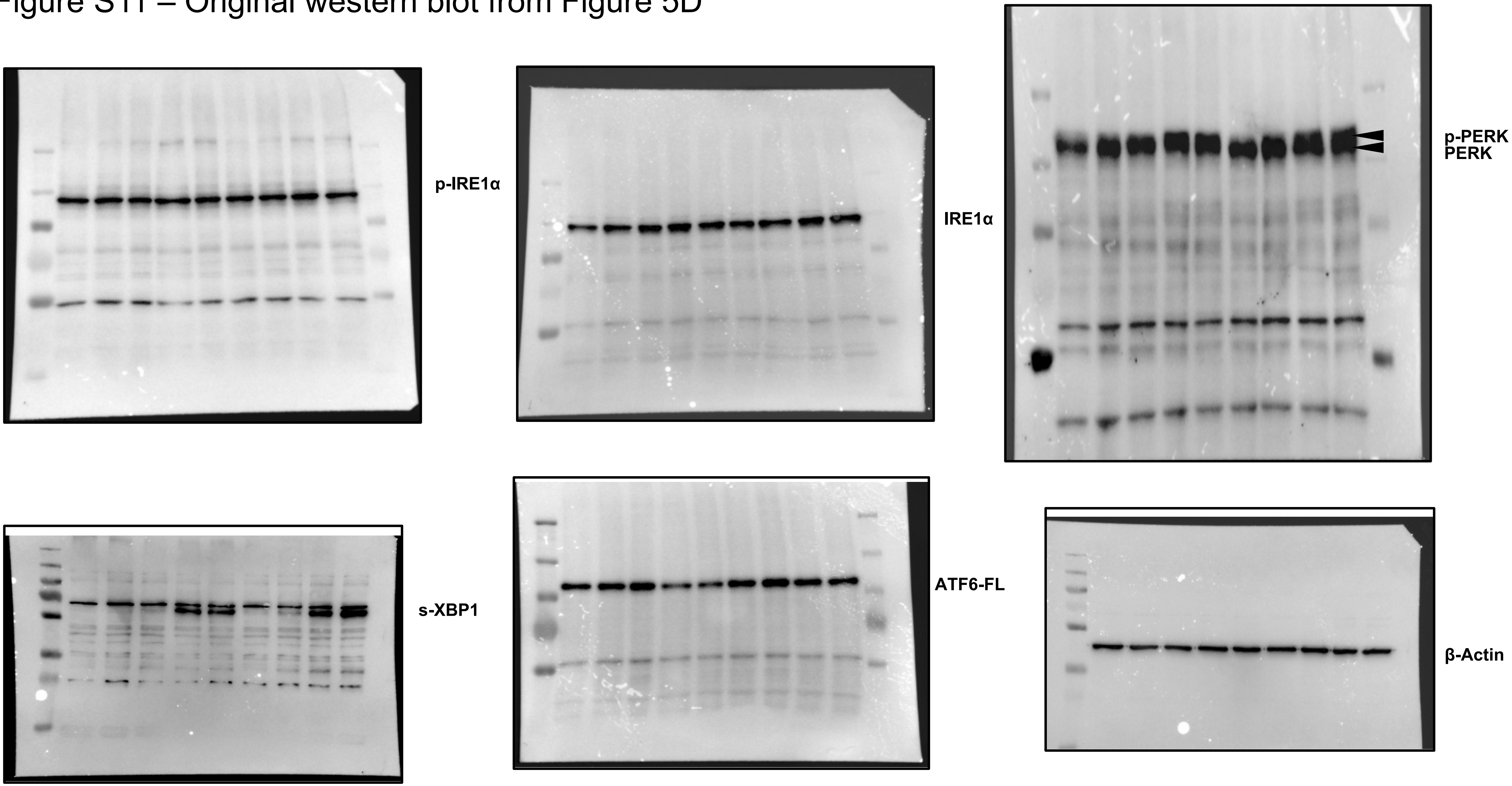

Figure S12 – Original western blot from Figure 6

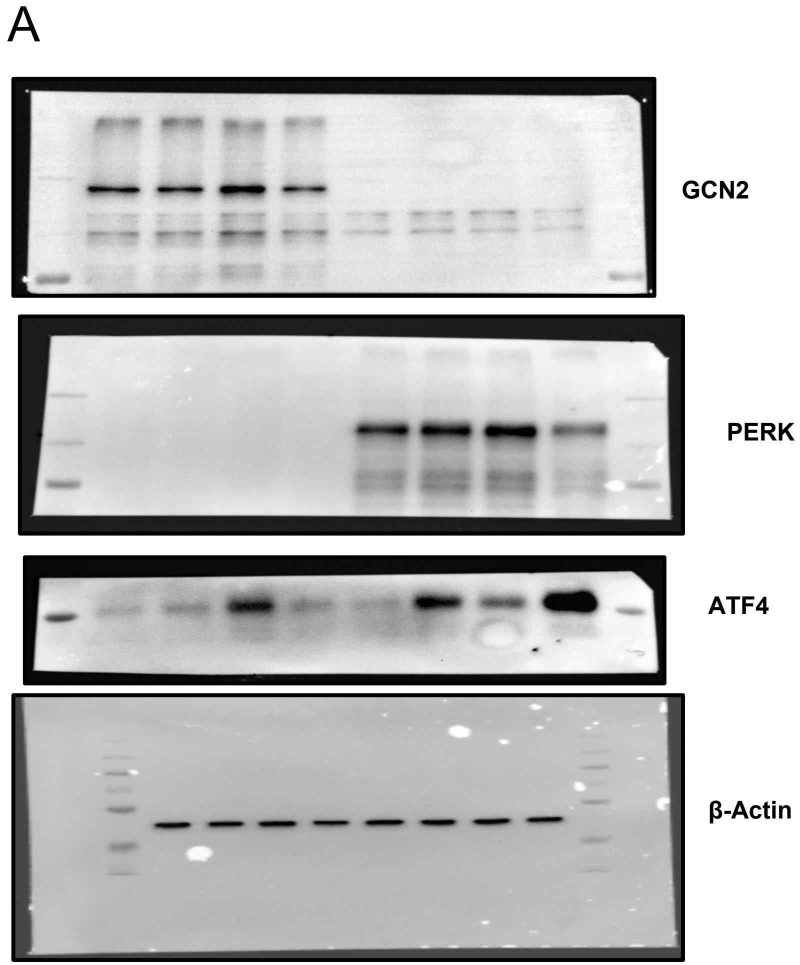

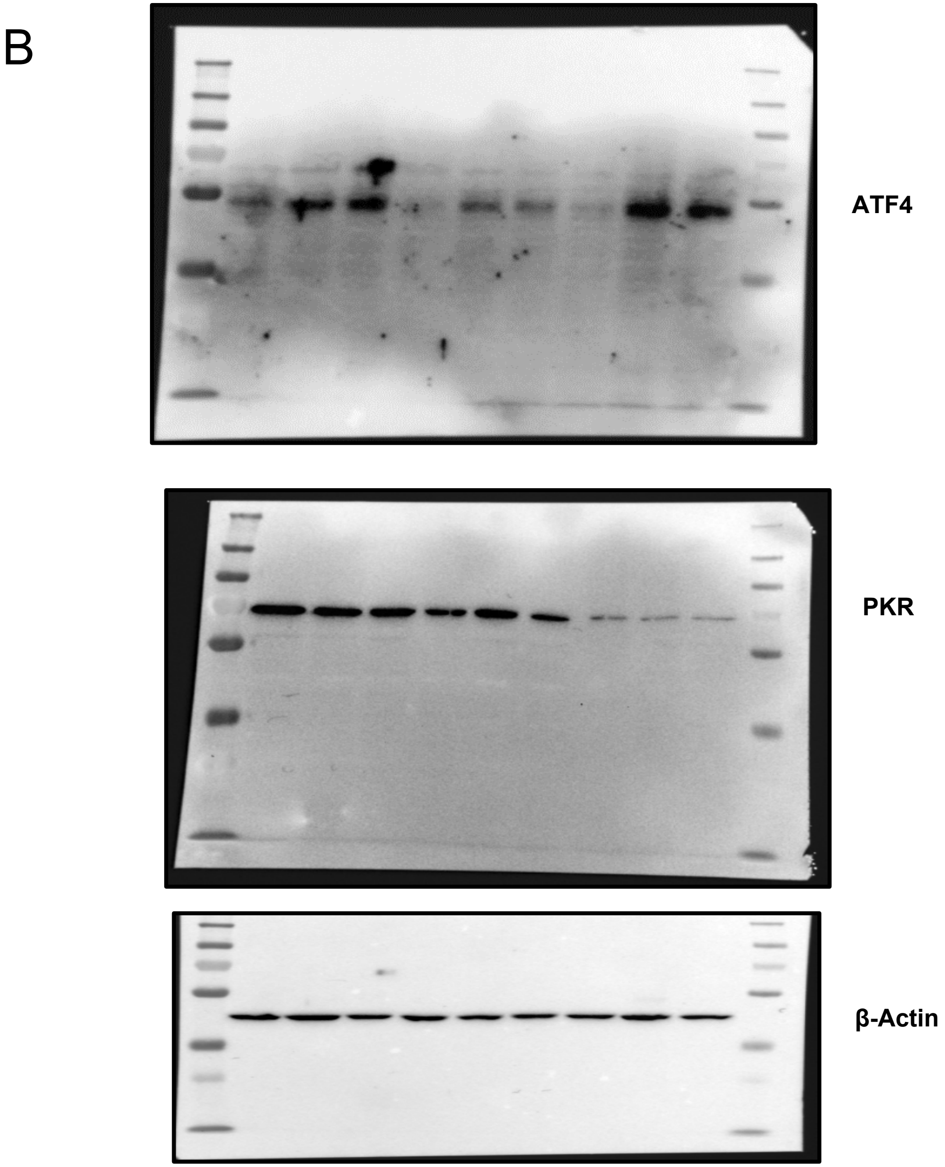

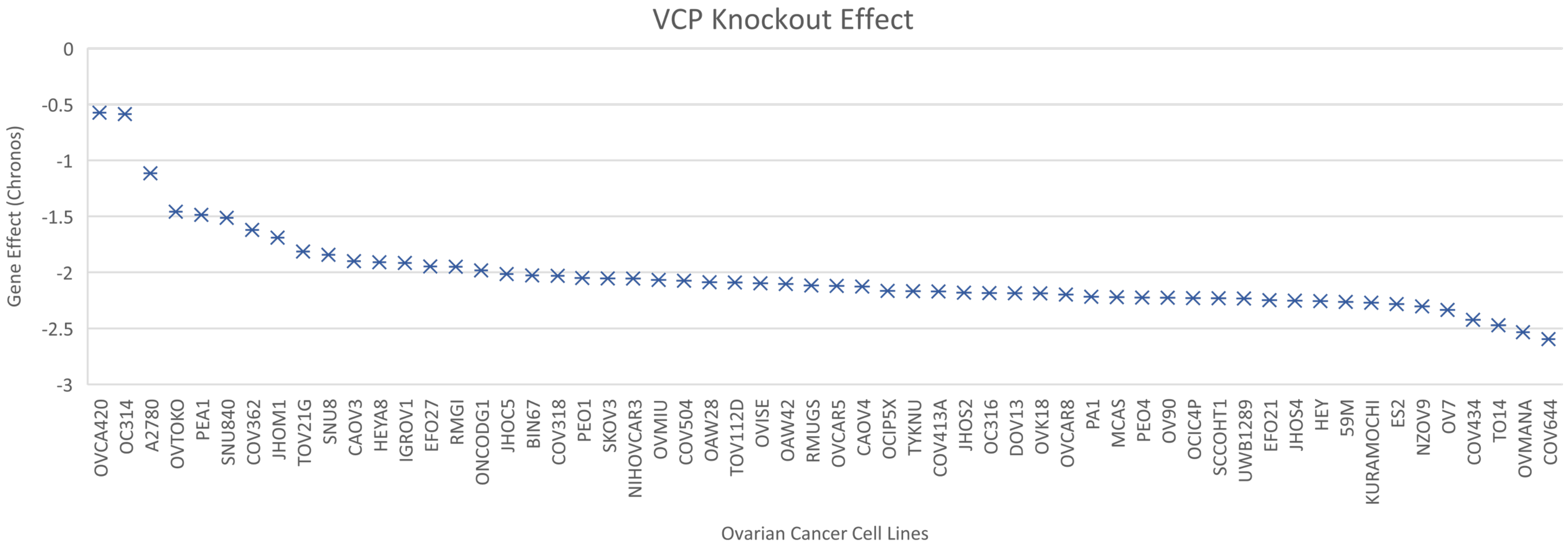

Figure S13. Effect of CRISPR/Cas9-mediated gene knockout of VCP in ovarian cancer cells. Negative gene effect indicates dependency. Gene effect < -1 Suggest strong dependency on the gene. Data is downloaded from the DepMap project and replotted with Microsoft Excel. COV318, ONCODG1, OVCAR3, and SNU8 are reported to have the CCNE1 amplification.

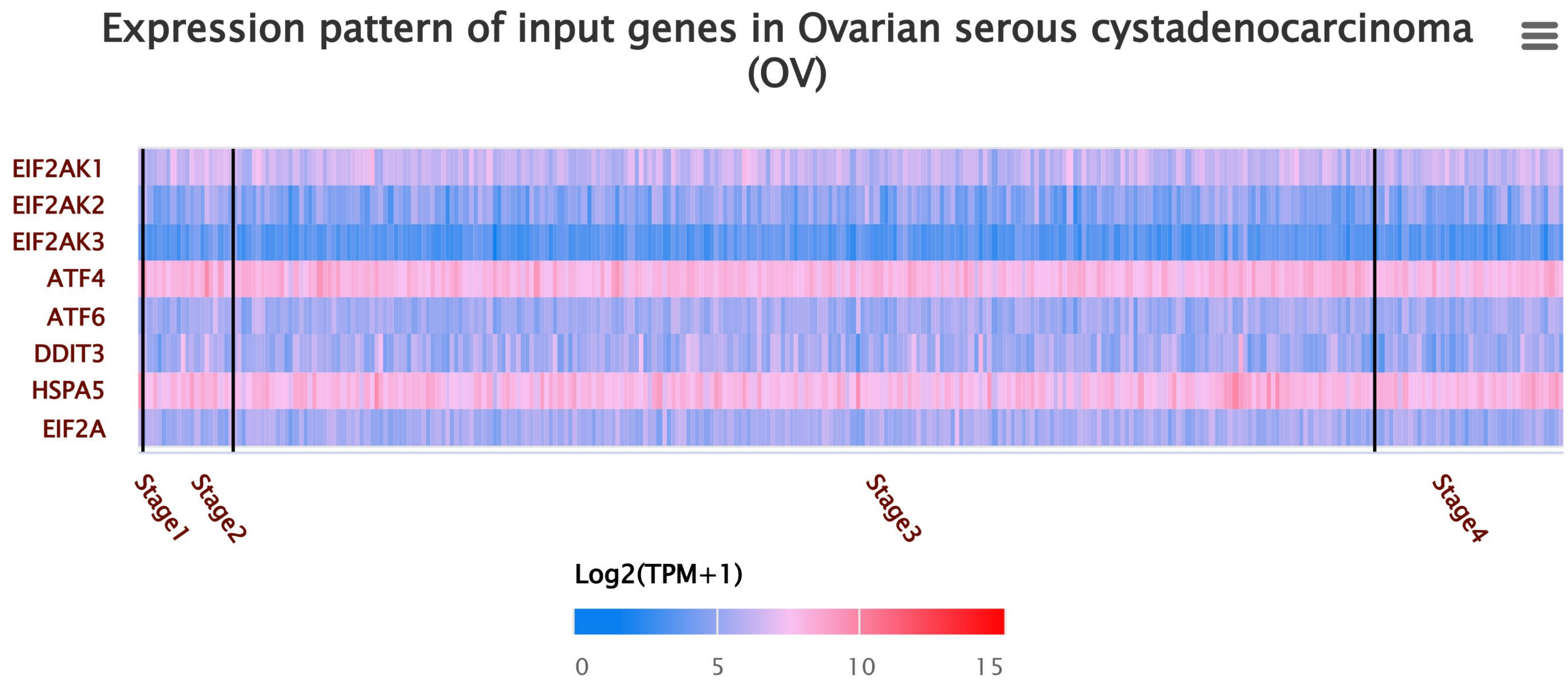

Figure S14 A: Analysis of the expression of select UPR-related genes in high-grade serous carcinomas from the TCGA dataset (from UALCAN).

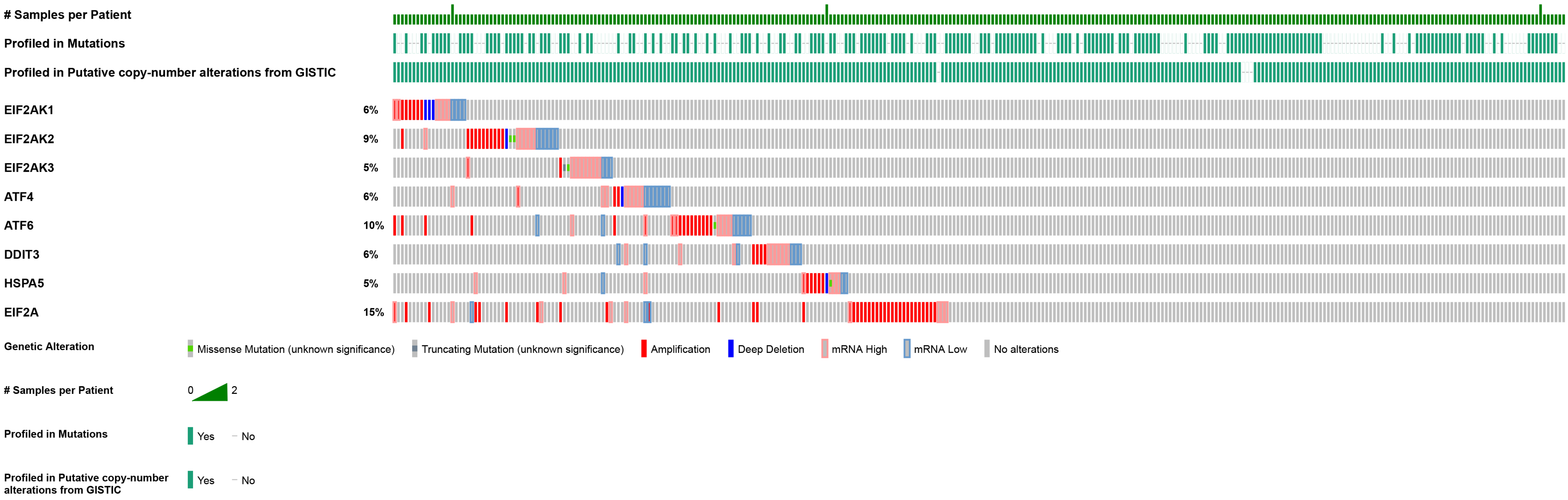

Figure S14 B. Analysis of genetic alterations in select UPR-related genes in high-grade serous carcinomas from the Cancer Genome Atlas dataset (from cbioportal).

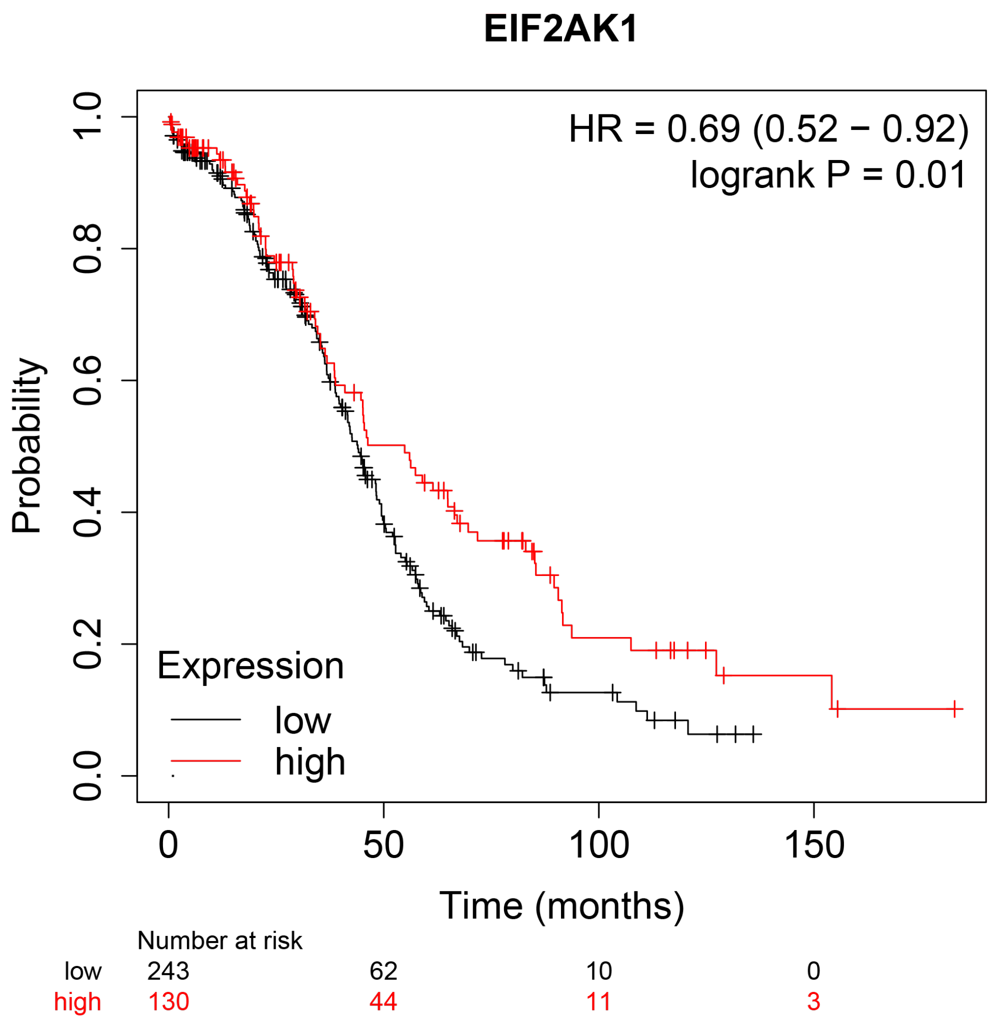

https://kmplot.com/analysis/index.php?p=view&pa_id=15382064&show=bZFNbo
QwDIXvwrqqNItuehnLBGewCHFkO8y0o969gUULiO33np__Xp3hQoAGzjF2n13EZNS
9dTbKA2YaGHOjrvUfcp7xeYI9kT1Q5-
sEkAhWdeEF07kQw9QKB1CKpJQDnSNKEofEmcBHDlMms2a5HWSnp0MvabhWjL_X
2Nv7x0EL1VxmmJFzW9_TdevShq4ERYydZbuGFOX76McJ6U552Nt68RafKK7GolLCS
GHa9fhjgCk98Mt2WjWCBZWxTwRRFIKMor63BFEFK9TOvj3pwFdsks8bheqy_Xn7wc
8v

Figure S14C

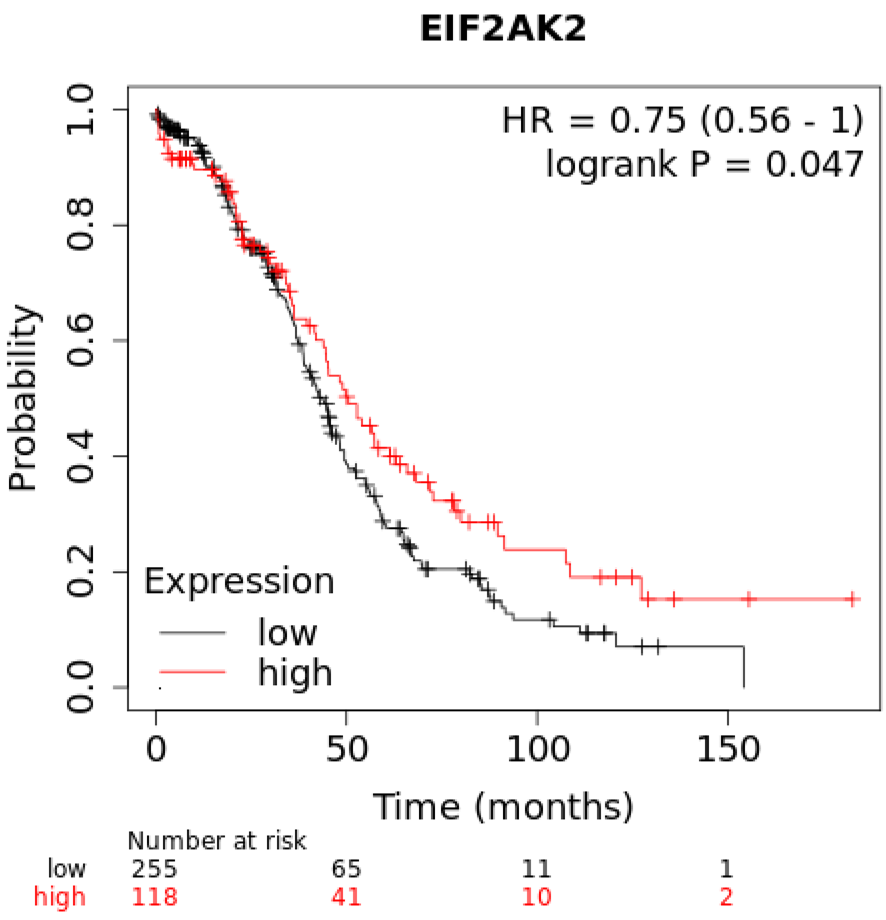

https://kmplot.com/analysis/index.php?p=view&pa_id=15382065&show=bZFNboQwDIXvwrqqNItuehnLBGewCHFkO8y0o969gUULiO33np__Xp3hQoAGzjF2n13EZNS9dTbKA2YaGHOjrvUfcp7xeYI9kT1Q5-sEkAhWdeEF07kQw9QKB1CKpJQDnSNKEofEmcBHDlMms2a5HWSnp0MvabhWjL_X2Nv7x0EL1VxmmJFzW9_TdevShq4ERYydZbuGFOX76McJ6U552Nt68RafKK7GolLCSGHa9fhjgCk98Mt2WjWCBZWxTwRRFIKMor63BFEFK9TOvj3pwFdsks8bheqy_Xn7wc8v

Figure S14C

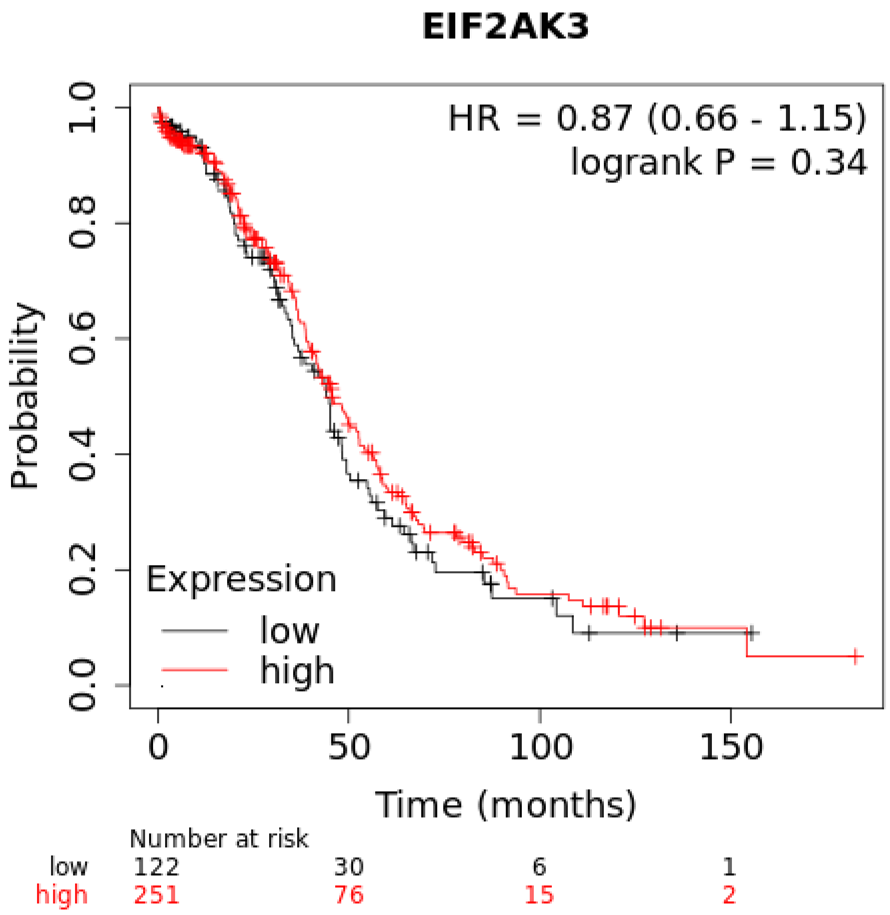

https://kmplot.com/analysis/index.php?p=view&pa_id=15382072&show=bZFNboQwDIXvwrqqNItuehnLBGewCHFkO8y0o969gUULiO33np__Xp3hQoAGzjF2n13EZNS9dTbKA2YaGHOjrvUfcp7xeYI9kT1Q5-sEkAhWdeEF07kQw9QKB1CKpJQDnSNKEofEmcBHDlMms2a5HWSnp0MvabhWjL_X2Nv7x0EL1VxmmJFzW9_TdevShq4ERYydZbuGFOX76McJ6U552Nt68RafKK7GolLCSGHa9fhjgCk98Mt2WjWCBZWxTwRRFIKMor63BFEFK9TOvj3pwFdsks8bheqy_Xn7wc8v

Figure S14C

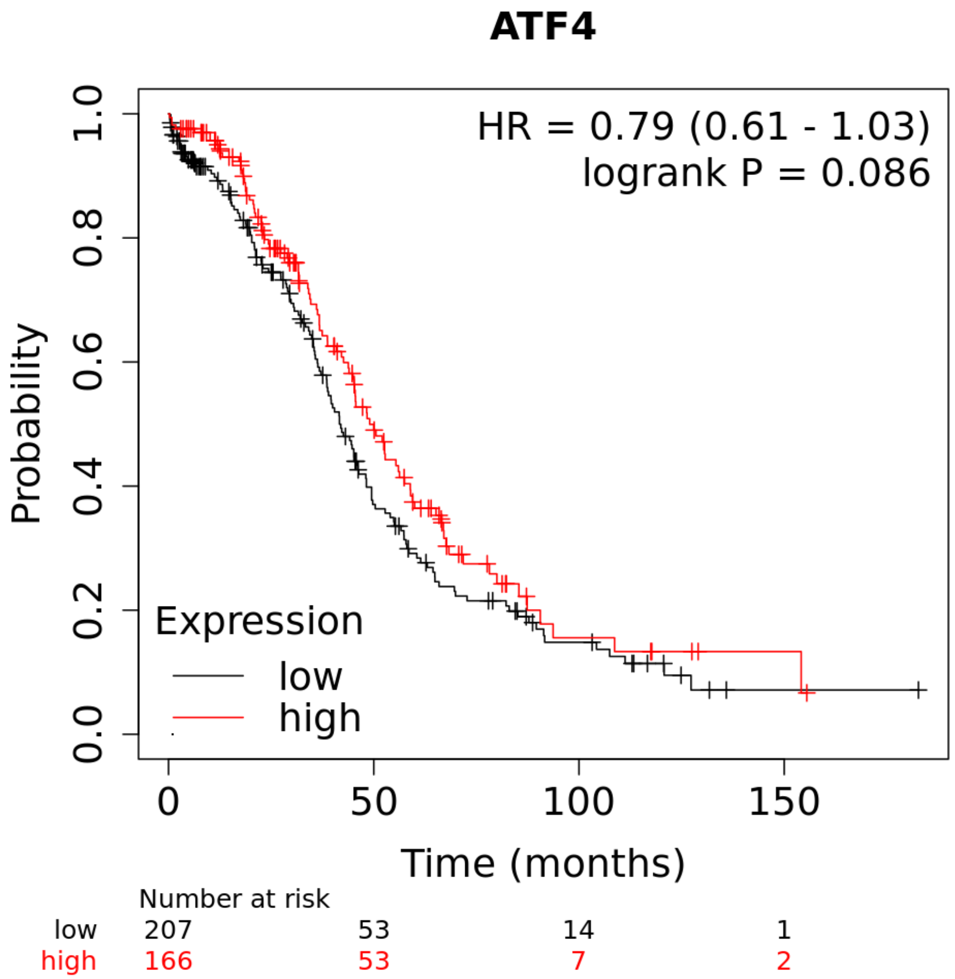

Figure S14C

https://kmplot.com/analysis/index.php?p=view&pa_id=15382062&show=bZFL
bsMwDETv4nVRIItuehmClqmYsCwKJOWkDXr3ylm0tpDtm-Hw9xgMNwI0cI5x-
BxcKw1vg81yg5UmxtxDziveOzgS2Q11bThisi4BJIJV3XjD1BdiWFrhBEqRlHKgPqIk
cUicCXzmsGQya5bLSXa6O4ySpteK8fcee3n_OGmhmssKK3Ju23t63bq0oStBEWN
neV5DivJ19vOEdKU8HW2jeItPFHdjUSlhprAcevwxwJRu-
GUHrRrBhso4JoIoCkFmUT9agqiCFWpnfz7pxHdskvuNQnX5f_PPLw

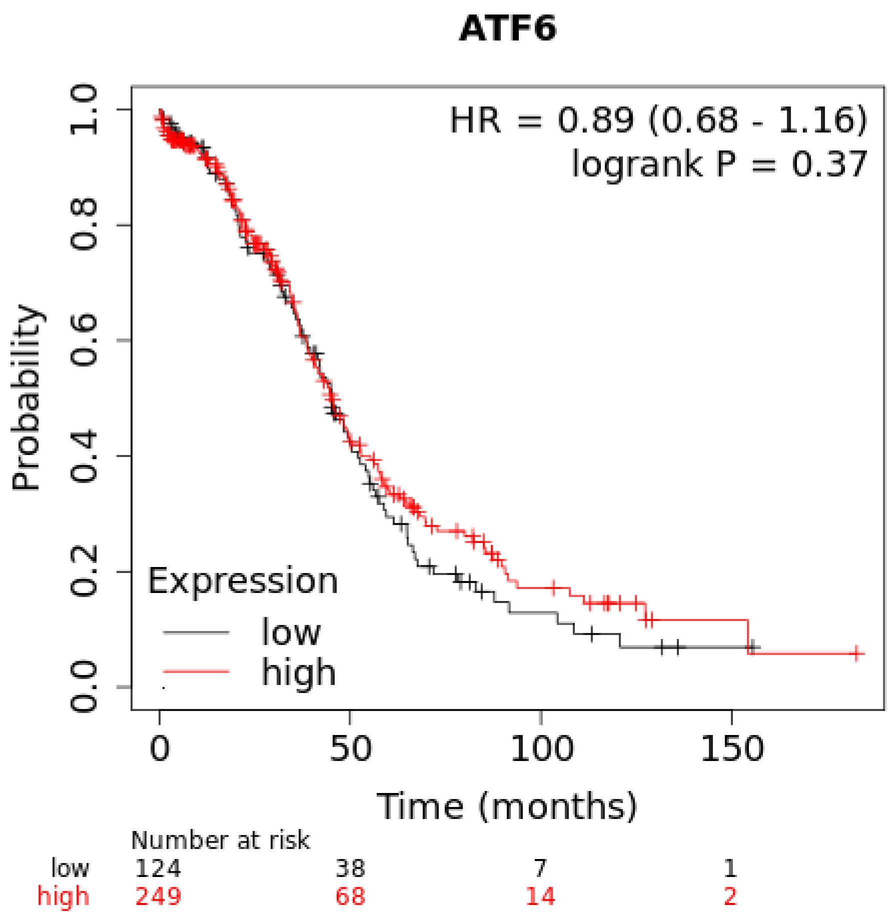

https://kmplot.com/analysis/index.php?p=view&pa_id=15382067&show=bZFNboQwDIXvwrqqNIt
uehnLBGewCHFkO8y0o969gUULiO33np__Xp3hQoAGzjF2n13EZNS9dTbKA2YaGHOjrvUfcp7xeYI9k
T1Q5-
sEkAhWdeEF07kQw9QKB1CKpJQDnSNKEofEmcBHDlMms2a5HWSnp0MvabhWjL_X2Nv7x0EL1Vxm
mJFzW9_TdevShq4ERYydZbuGFOX76McJ6U552Nt68RafKK7GolLCSGHa9fhjgCk98Mt2WjWCBZWxT
wRRFIKMor63BFEFK9TOvj3pwFdsks8bheqy_Xn7wc8v

Figure S14C

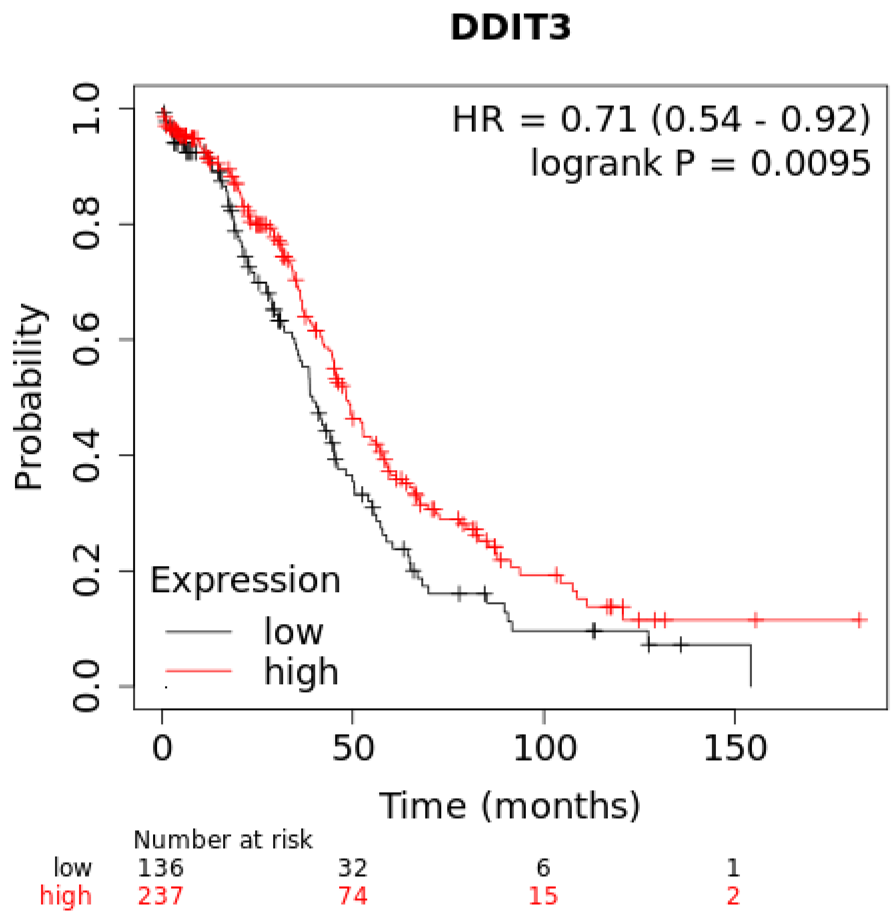

https://kmplot.com/analysis/index.php?p=view&pa_id=15382068&show=bZFNboQwDIXvwrqqNItuehnLBGewCHFkO8y0o969gUULiO33np__Xp3hQoAGzjF2n13EZNS9dTbKA2YaGHOjrvUfcp7xeYI9kT1Q5-sEkAhWdeEF07kQw9QKB1CKpJQDnSNKEofEmcBHDlMms2a5HWSnp0MvabhWjL_X2Nv7x0EL1VxmmJFzW9_TdevShq4ERYydZbuGFOX76McJ6U552Nt68RafKK7GolLCSGHa9fhjgCk98Mt2WjWCBZWxTwRRFIKMor63BFEFK9TOvj3pwFdsks8bheqy_Xn7wc8v

Figure S14C

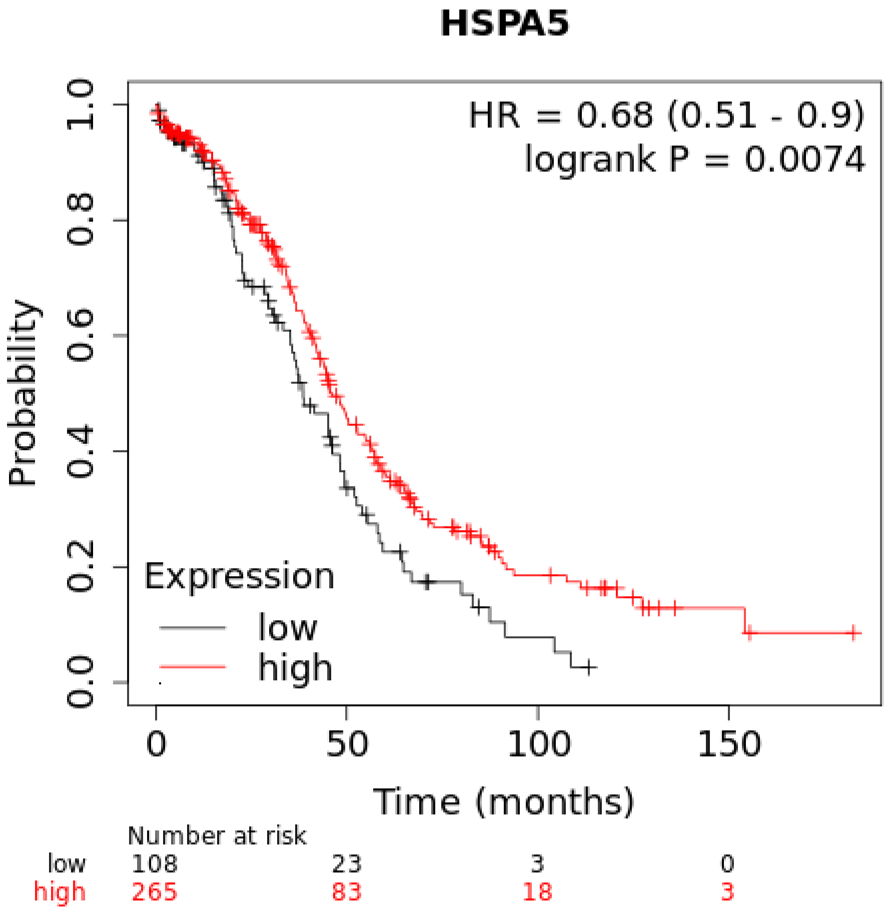

https://kmplot.com/analysis/index.php?p=view&pa_id=15382069&show=bZFNboQwDIXvwrqqN
ItuehnLBGewCHFkO8y0o969gUULiO33np__Xp3hQoAGzjF2n13EZNS9dTbKA2YaGHOjrvUfcp7xeYI
9kT1Q5-
sEkAhWdeEF07kQw9QKB1CKpJQDnSNKEofEmcBHDlMms2a5HWSnp0MvabhWjL_X2Nv7x0EL1Vx
mmJFzW9_TdevShq4ERYydZbuGFOX76McJ6U552Nt68RafKK7GolLCSGHa9fhjgCk98Mt2WjWCBZ
WxTwRRFIKMor63BFEFK9TOvj3pwFdsks8bheqy_Xn7wc8v

Figure S14C

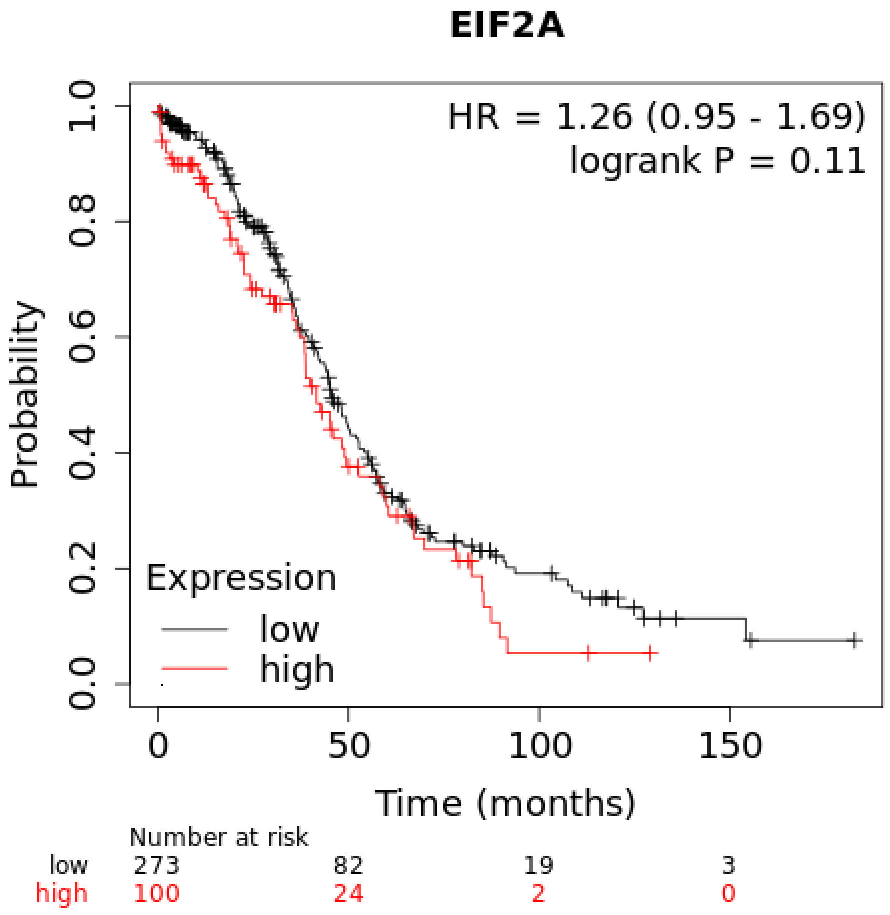

https://kmplot.com/analysis/index.php?p=view&pa_id=15382070&show=bZFNboQwDIXvwrqq
NItuehnLBGewCHFkO8y0o969gUULiO33np__Xp3hQoAGzjF2n13EZNS9dTbKA2YaGHOjrvUfcp7x
eYI9kT1Q5-
sEkAhWdeEF07kQw9QKB1CKpJQDnSNKEofEmcBHDlMms2a5HWSnp0MvabhWjL_X2Nv7x0EL1V
xmmJFzW9_TdevShq4ERYydZbuGFOX76McJ6U552Nt68RafKK7GolLCSGHa9fhjgCk98Mt2WjWC
BZWxTwRRFIKMor63BFEFK9TOvj3pwFdsks8bheqy_Xn7wc8v

Figure S14C

Figure S14 C. Overall survival analysis of select genes in high-grade serous carcinomas obtained from the Kaplan-Meier Plotter using the RNAseq dataset from the Pancancer survival analysis. Auto select best cutoff option is used to dichotomize into low and high expression groups. Each cutoff value for each plot can be viewed in permanent weblink provided next to the plot.
